# Oral Health Conditions and Dental Visits Among Pregnant and Nonpregnant Women of Childbearing Age in the United States, National Health and Nutrition Examination Survey, 1999–2004

**DOI:** 10.5888/pcd11.140212

**Published:** 2014-09-18

**Authors:** Alejandro Azofeifa, Lorraine F. Yeung, C. J. Alverson, Eugenio Beltrán-Aguilar

**Affiliations:** Author Affiliations: Lorraine F. Yeung, C. J. Alverson, Eugenio Beltrán-Aguilar, Centers for Disease Control and Prevention, Atlanta, Georgia.

## Abstract

**Introduction:**

Oral diseases can be prevented or improved with regular dental visits. Our objective was to assess and compare national estimates on self-reported oral health conditions and dental visits among pregnant women and nonpregnant women of childbearing age by using data from the National Health and Nutrition Examination Survey (NHANES).

**Methods:**

We analyzed self-reported oral health information on 897 pregnant women and 3,971 nonpregnant women of childbearing age (15–44 years) from NHANES 1999–2004. We used χ^2^ and 2-sample *t* tests to assess statistical differences between groups stratified by age, race/ethnicity, poverty, and education. We applied the Bonferroni adjustment for multiple comparisons.

**Results:**

Our data show significant differences in self-reported oral health conditions and dental visits among women, regardless of pregnancy status, when stratified by selected sociodemographic characteristics. Significant differences were also found in self-reported oral health conditions and dental visits between pregnant and nonpregnant women, especially among young women, women from minority race/ethnicity groups, and women with less than high school education.

**Conclusion:**

We found disparities in self-reported oral health conditions and use of dental services among women regardless of pregnancy status. Results highlight the need to improve dental service use among US women of childbearing age, especially young pregnant women, those who are non-Hispanic black or Mexican American, and those with low family income or low education level. Prenatal visits could be used as an opportunity to encourage pregnant women to seek preventive dental care during pregnancy.

## Introduction

Oral diseases such as dental caries, gingivitis, and chronic adult periodontitis are common and may cause pain and disability in all age groups and in vulnerable individuals, including pregnant women. Studies suggest that intraoral infections may be associated with adverse pregnancy outcomes (1–5). However, there is no conclusive evidence on these associations (6,7). Some adverse consequences of oral diseases (eg, tooth loss, pain) can be avoided by early treatment during regular dental visits. One of the objectives of *Healthy People 2020* is to increase the proportion of children, adolescents, and adults who used the oral health care system in the previous 12 months (8). A study using data from the National Health and Nutrition Examination Survey (NHANES, 1999–2004) indicated that 64% of women aged 20 to 64 years reported a dental visit in the previous year (9). However, no national estimates of dental visits based on those NHANES data were reported for pregnant women or women of childbearing age. Studies from the Pregnancy Risk Assessment Monitoring System (PRAMS) showed that 23% to 44% of women with a recent live birth sought dental care during pregnancy (10–12), and studies from the Behavioral Risk Factor Surveillance System (BRFSS) showed that 70% of pregnant women received dental care in the previous 12 months (13). None of these studies provided estimates for women of childbearing age or compared the differences between pregnant and nonpregnant women. There is also a lack of information on national estimates of the self-reported oral health of pregnant and nonpregnant women. The objective of this study was to assess the national prevalence of self-reported oral health conditions and dental visits among US pregnant women and nonpregnant women of childbearing age (15–44 years) and to compare the differences in prevalence between the 2 groups of women by using data from NHANES (1999–2004).

## Methods

We used data from NHANES, an ongoing, complex, multistage survey designed to estimate the nutrition and health status of the noninstitutionalized, civilian US population. NHANES data are collected via household interviews and physical examinations at the Mobile Examination Center. Detailed information regarding NHANES is available on the NHANES website (14). Although NHANES oversampled pregnant women from 1999 through 2006, the question about conditions of mouth and teeth was asked only from 1999 through 2002, and questions about use of dental services were asked only from 1999 through 2004. Responses to these questions were obtained from a face-to-face standardized household interview conducted at the participants’ home.

We analyzed the responses to 3 oral health questions from the household interview in the 1999 through 2004 survey cycle: 1) “How would you describe the condition of your mouth and teeth?” (data were available from 1999 through 2002 only); we combined responses “very good” and “good” into 1 category to reflect a positive perceived oral health condition of the participants’ mouth and teeth and to increase statistical power; 2) “About how long has it been since you last visited a dentist?” (data were available from 1999 through 2004); we combined responses “6 months or less” and “more than 6 months, but not more than 1 year ago” into 1 category to ascertain how many participants had a dental visit in the previous year; and 3) “What was the main reason you last visited the dentist?” (data were available from 1999 through 2004); we selected the following response, “went in on their own for check-up, examination, or cleaning” to denote a preventive dental visit. We first analyzed data on pregnant women and nonpregnant women of childbearing age (15–44 years) separately and then compared the differences in estimates between the 2 groups. We used the pregnancy variable provided by NHANES (RIDEXPREG) to select pregnant women (14). According to NHANES, pregnancy status was ascertained through either the interview question “are you currently pregnant?” or a urine pregnancy test conducted at examination. Detailed information regarding how pregnancy status is determined in the survey is available at the NHANES website (14). We used data on 622 pregnant women and 2,561 nonpregnant women of childbearing age in the analysis of question 1, condition of mouth and teeth; 193 women were excluded because their pregnancy status could not be ascertained. This oral health question was discontinued after survey cycle 2002. We used data on 897 pregnant women and 3,971 nonpregnant women of childbearing age in the analysis of questions 2 and 3 about dental visits; we excluded 265 women because their pregnancy status could not be ascertained. We stratified our analysis by age, race/ethnicity, poverty status, and educational level. Age was categorized into 3 groups: 15 to 24 years, 25 to 34 years, and 35 to 44 years. We included 3 racial/ethnic groups: non-Hispanic white, non-Hispanic black, and Mexican American. Other racial/ethnic groups, such as other Hispanics and other race (which includes multiracial) were not reported separately because of small sample size but were included in the denominators in estimations of prevalence. Poverty status was defined by the ratio of family income to the federal poverty level (FPL), and we included 3 poverty categories: <100% of the FPL, 100% to 199% of the FPL, and 200% or more of the FPL. We included 3 education categories provided by NHANES: less than high school, high school diploma, and more than high school.

We used SAS version 9.3 (SAS Institute, Inc) and SUDAAN version 10.0 (Research Triangle Institute) for analysis to account for the complex sampling design. We used 6 years (1999–2004) and 4 years (1999–2002) sample Mobile Examination Center weights to produce accurate population estimates. Detailed information for the survey sample weights and the NHANES analytical guidelines are available from the NHANES website (14). Tabular distributions of sociodemographic characteristics are presented and contrasted by pregnancy status using χ2 tests at the α = 0.05 level (Table 1). Percentage estimates for selected response categories for oral health questions are presented and contrasted by pregnancy status using 95% confidence interval (CI) and 2-sample *t* tests (Table 2 and Table 3). All significant comparisons in estimates (*P* < .05) are indicated in Table 2 and Table 3. We further applied the Bonferroni adjustment for multiple comparisons and included in “Results” only comparisons that remained significantly different after the Bonferroni adjustment. For the 13 pairwise tests between pregnant and nonpregnant women, the differences are significant if the Bonferroni *P* value is less than .004; and for the 16 pairwise tests between respondent characteristic groups, the differences are significant if the Bonferroni *P* value is less than .003. We indicated in the tables when an estimate had degrees of freedom less than 12. *P* values presented in the text are averaged and may differ from the tables.

**Table 1:** Sociodemographic Characteristics of Pregnant Women (N = 897) and Nonpregnant Women of Childbearing Age (N = 3,971), National Health and Nutrition Examination Survey, 1999–2004

Selected Characteristic	Pregnant Women, % (SE)	Nonpregnant women, % (SE)	*P* Value^a^
**Age, y**			
15–24	34.0 (2.46)	30.4 (0.98)	<.001
25–34	52.4 (2.77)	30.4 (1.18)	
35–44	13.6 (2.26)	39.2 (1.31)	
**Race/ethnicity** b			
Non-Hispanic white	54.0 (3.43)	66.2 (1.96)	.05
Non-Hispanic black	16.5 (2.29)	13.0 (1.22)	
Mexican American	14.4 (1.85)	9.2 (1.02)	
**Poverty status (% FPL)** c			
<100%	23.4 (2.85)	20.3 (1.14)	.144
100%–199%	18.2 (2.32)	22.1 (0.93)	
≥200%	58.4 (3.96)	57.6 (1.38)	
**Education**			
<High school	22.9 (2.36)	24.0 (0.88)	.126
High school diploma	19.1 (2.33)	23.0 (1.03)	
≥High school	58.0 (2.83)	52.9 (1.34)	
**Marital status**			
Married	62.9 (3.05)	44.5 (1.18)	<.001
**Covered by health insurance**	85.7 (1.74)	78.0 (1.15)	.05
**Covered for dental care** d	76.6 (2.90)	77.0 (1.17)	.876

Abbreviations: SE, standard error; FPL, federal poverty level.

a χ^2^ test was used to compare estimates between pregnant and nonpregnant women for the distribution of selected sociodemographic characteristics.

b All race/ethnicity categories are included in the total but not presented as separate race/ethnicity strata.

c Poverty status is the ratio of family income to the federal poverty level.

d Coverage was determined by answer to question: “Does the insurance (you have) cover any part of dental care?” Data were available only for NHANES survey cycle 1999–2002.

**Table 2:** Self-Assessment of Oral Health Conditions of Pregnant Women (N = 622) and Nonpregnant Women of Childbearing Age (N = 2,561) by Selected Characteristics, National Health and Nutrition Examination Survey, 1999–2002

Selected Characteristic	Having Very Good or Good Mouth and Teeth Condition		
	Pregnant Women, % (95% CI)	Nonpregnant Women, % (95% CI)	*P* Value^a^
**Total sample**	66.9 (61.1–72.7)	70.1 (67.7–72.5)	.322
**Age, y**			
15–24	57.2 (45.0–69.5)^y^	75.3 (71.8–78.8)^y^	.005
25–34	67.9 (59.4–76.5)^z^	69.1 (65.5–72.7)	.801
35–44	85.8 (76.0–95.6)^b^^,^ c ^,^ d ^,y,z^	67.0 (63.3–70.8)^y^	<.001
**Race/ethnicity** e			
Non-Hispanic white	77.1 (69.7–84.5)^y,z^	74.4 (70.6–78.2)^y,z^	.524
Non-Hispanic black	46.2 (29.5–63.0)^y^	62.9 (58.9–66.9)^y^	.035
Mexican American	37.5 (23.9–51.1)^z^	47.7 (43.0–52.4)^z^	.106
**Poverty status (% FPL)** f			
<100	51.7 (40.9–62.6)^y^	54.0 (48.7–59.4)^y^	.652
100–199	55.3 (40.0–70.7)^b^	59.1 (52.7–65.6)^z^	.664
≥200	77.0 (70.0–83.9)^y,^ b	81.1 (78.1–84.1)^y,z^	.303
**Education**			
<High school	51.7 (39.4–64.0)^y^	57.2 (52.8–61.6)^y^	.437
High school diploma	43.7 (28.2–59.2)^z^	61.7 (56.5–66.9)^z^	.02
≥High school	81.1 (73.8–88.5)^y,z^	79.7 (76.4–82.9)^y,z^	.746

Abbreviations: CI, confidence interval; FPL, federal poverty level.

a *P* value compares estimates between pregnant and nonpregnant women in each category (eg, age, race/ethnicity). A total of 13 pairwise tests between pregnant and nonpregnant women, yielding a Bonferroni corrected cut-off of .05/13 (*P* = <.004). *P* values for these tests exceeding .004 should be interpreted with caution on the basis of Bonferroni adjustment for multiple comparisons.

b Two sample *t* tests were used to compare estimates in each category (eg, age, race/ethnicity) for both pregnant and nonpregnant women. *P* value is greater than .003 but less than .05.

c *P* value compares estimates in each category (eg, age, race/ethnicity) for pregnant and nonpregnant women. A total of 16 pairwise tests between respondent characteristic groups yielded a Bonferroni corrected cut-off of .05/16, *P* = <.003. *P* values for these tests exceeding .003 should be interpreted with caution on the basis of Bonferroni adjustment for multiple comparisons. Values in a sociodemographic subgroup (eg, age groups) among either pregnant or nonpregnant women with the same superscript letters (y and z) mean they are significantly different from one another, *P* < .003 (eg, If the percentage of pregnant women aged 15 to 24 years who answered “yes” to having very good or good mouth and teeth condition is significantly different from the percentage of pregnant women aged 35 to 44 who answered “yes” to having very good or good mouth and teeth condition, they will both have the same superscript letter.

d Estimate may not be representative (degrees of freedom = 11).

e All racial/ethnic categories are included in the total denominator but not all racial/ethnic categories are presented separately.

f Poverty status is defined by the ratio of family income to the federal poverty level.

**Table 3:** Self-Assessment of Dental Visits of Pregnant Women (N = 897) and Nonpregnant Women of Childbearing Age (N = 3,971) by Selected Characteristics, National Health and Nutrition Examination Survey, 1999–2004

Characteristic	Having a Dental Visit in Previous Year			Having Preventive Care as the Main Reason for Last Dentist Visit		
	Pregnant Women, % (95% CI)	Nonpregnant Women, % (95% CI)	*P* Value^a^	Pregnant Women, % (95% CI)	Nonpregnant Women, % (95% CI)	*P* Value^a^
**Total sample**	58.3 (51.8–64.8)	64.8 (62.7–66.8)	.040	61.4 (54.3–68.4)	55.9 (53.4–58.4)	.131
**Age, y**						
15–24	51.0 (41.6–60.4)^b^	65.6 (62.5–68.8)	.003	55.7 (47.4–63.9)	58.0 (54.2–61.9)	.570
25–34	58.6 (50.6–66.6)^c^	63.8 (60.6–67.0)	.178	62.9 (52.5–73.2)	56.3 (53.5–59.1)	.228
35–44	75.3 (60.7–89.9)^b^^,^ c ^,^ d	64.8 (61.4–68.3)	.133	69.4 (50.9–88.0)	54.0 (50.0–57.9)	.08
**Race/ethnicity** e						
Non-Hispanic white	71.1 (62.5–79.7)^y,z^	68.9 (66.1–71.7)^y,z^	.624	67.6 (57.9–77.2)^c^^,^ d	56.7 (53.5–60.0)	.028
Non-Hispanic black	39.5 (30.7–48.3)^y^	58.2 (53.5–62.9)^y^	<.001	53.3 (41.9–64.7)^c^	54.4 (50.4–58.4)	.840
Mexican American	29.9 (21.9–37.9)^z^	47.1 (41.9–52.2)^z^	<.001	51.5 (40.4–62.7)^d^	51.8 (48.1–55.5)	.961
**Poverty status (% FPL)** ^f^						
<100	40.6 (28.4–52.8)^y^	52.9 (47.7–58.2)^y^	.037	41.4 (27.3–55.5)^y^	42.7 (38.2–47.2)^y^	.862
100–199	54.1 (43.3–64.9)	51.4 (45.8–57.1)^z^	.662	55.1 (39.5–70.7)	46.3 (41.1–51.5)^z^	.306
≥200	66.2 (58.4,73.9)^y^	74.1 (71.3–76.8)^y,z^	.048	70.1 (60.7–79.4)^y^	64.4 (61.9–66.9)^y,z^	.242
**Education level**						
<High school	41.0 (32.6–49.3)^y^	56.0 (52.5–59.6)^y^	<.001	53.1 (40.5–65.7)^b^	47.4 (43.6–51.2)^y^	.373
High school diploma	48.0 (37.0–59.0)^z^	57.6 (53.2–62.1)^z^	.124	48.5 (34.2–62.7)^c^	48.0 (44.2–51.8)^z^	.945
≥High school	68.6 (60.7–76.4)^y,z^	71.9 (68.8–75.1)^y,z^	.350	68.5 (59.7–77.3)^b^^,^ c	63.1 (59.4–66.7)^y,z^	.243

Abbreviations: CI, confidence interval; FPL, federal poverty level.

a *P* value compares estimates between pregnant and nonpregnant women in each category (eg, age, race/ethnicity). A total of 13 pairwise tests between pregnant and nonpregnant women yielded a Bonferroni corrected cut-off of .05/13, *P* = <.004. *P* values for these tests exceeding .004 should be interpreted with caution on the basis of Bonferroni adjustment for multiple comparisons.

b,c Two sample *t* tests were used to compare estimates in each category (eg, age, race/ethnicity) in both pregnant and nonpregnant women. *P* value is greater than .003 but less than .05.

d *P* value compares estimates in each category (eg, age, race/ethnicity) in both pregnant and nonpregnant women. A total of 16 pairwise tests between respondent characteristic groups yielded a Bonferroni corrected cut-off of .05/16, *P* = <.003. *P* values for these tests exceeding .003 should be interpreted with caution on the basis of a Bonferroni adjustment for multiple comparisons. Values in a sociodemographic subgroup (eg, age groups) among either pregnant or nonpregnant women with the same superscript letters (y and z) are significantly different from one another *P* < .003 (eg, if the percentage of pregnant women aged 15 to 24 years who answered “yes” to having a dental visit in the previous year is significantly different than the percentage of pregnant women aged 35–44 who answered “yes” to having a dental visit in the previous year, they will both have the same superscript letter.

e All racial/ethnic categories are included in the total denominator, but not all racial/ethnic categories are presented separately.

f Poverty status is defined by the ratio of family income to the federal poverty level.

## Results

### Sociodemographic characteristics

Pregnant women differed significantly in age and race/ethnicity from nonpregnant women (overall *P* < .05) (Table 1). There were more pregnant than nonpregnant women in the 25-to-34 age group but fewer pregnant than nonpregnant women in the 35-to-44 age group. Also, pregnant Mexican American women outnumbered nonpregnant Mexican American women. A higher proportion of pregnant than nonpregnant women were married and covered by health insurance (overall *P* < .05).

### Having very good or good mouth and teeth condition

The percentage of women who reported having very good or good mouth and teeth condition was significantly higher among older pregnant women (aged 35–44 years) than among younger pregnant women (15–24 years, 85.8% vs 57.2%, *P* = .002; 25–34 years, 85.8% vs 67.9%, *P* < .001( Table 2). In contrast, the percentage of women who reported having very good or good mouth and teeth condition was significantly higher among younger nonpregnant women aged 15 to 24 than among older pregnant women aged 35 to 44 (75.3% vs 67.0%, *P* = .003).

For both pregnant and nonpregnant women, significantly higher percentages of non-Hispanic white women (77.1% of pregnant women and 74.4% of nonpregnant women), women with family income at or more than 200% of the FPL (77.0% of pregnant women and 81.1% of nonpregnant women), and women with more than high school education (81.1% of pregnant women and 79.7% of nonpregnant women) reported having very good or good mouth and teeth condition than did those in other racial/ethnic groups (overall *P* ≤ .001), those with incomes less than 100% of the FPL (all with *P* < .001), those with incomes between 100% and 199% of the FPL (only nonpregnant women with *P* < .001) and low education levels (all with *P* < .001) (Table 2). A higher percentage of pregnant women aged 35 to 44 years reported their mouth and teeth condition being very good or good than did nonpregnant women of the same age (85.8% vs 67.0%, *P* < .001) (Table 2).

### Having a dental visit in the previous year

For both pregnant and nonpregnant women, significantly higher percentages of non-Hispanic whites (71.1% of pregnant women and 68.9% of nonpregnant women) and those with more than high school education (68.6% of pregnant women and 71.9% of nonpregnant women) reported having a dental visit in the previous year compared with other racial/ethnic and education groups (all with *P* < .001) (Table 3). A higher percentage of pregnant women with family income greater than 200% of the FPL reported having a dental visit in the previous year compared with those with family income less than 100% FPL (66.2% vs 40.6%, *P* < .001). A higher percentage of nonpregnant women with family income greater than 200% of the FPL reported having a dental visit in the previous year compared with nonpregnant women with lower incomes (74.1% vs 52.9% for those with <100% FPL and 74.1% vs 51.4%, for those with 100%−199% FPL; *P* < .001 for both).

A lower percentage of pregnant women aged 15–24 years reported having dental visits in the previous year than nonpregnant women of same age group (51.0% vs 65.6%, *P* = .003, Table 3). A lower percentage of non-Hispanic black and Mexican American pregnant women reported having a dental visit in the previous year compared with their nonpregnant counterparts (39.5% vs 58.2% for non-Hispanic blacks, *P* < .001; and 29.9% vs 47.1% for Mexican Americans, *P* < .001). A lower percentage of pregnant women having less than a high school education reported a dental visit in the previous year compared with nonpregnant women of the same educational level (41.0% vs 56.0%, *P* < .001).

### Having preventive care as the main reason for last dental visit

A higher percentage of pregnant women with family income greater than 200% of the FPL reported having preventive care as the main reason for their last dental visit than did those with family income less than 100% of the FPL (70.1% vs 41.4%, *P* < .001) (Table3). Likewise, a higher percentage of nonpregnant women with family income greater than 200% of the FPL reported having preventive care as the main reason for their last dental visit than did those with lower family incomes (64.4% vs 42.7% for <100% FPL and 64.4% vs 46.3% for 100%−199% FPL, *P* < .001 for both). A higher percentage of nonpregnant women with more than high school education reported having preventive care as the main reason for their last dental visit than did those with less education (63.1% vs 47.4% for less than high school and 63.1% vs 48.0% for those with high school diploma, *P* < .001 for both).

## Discussion

This study is the first to provide national estimates on self-reported oral health conditions and dental visits of pregnant women and nonpregnant women of childbearing age and to compare the differences in estimates between the 2 groups. We found differences among women stratified by selected sociodemographic characteristics regardless of pregnancy status. In general, non-Hispanic black and Mexican American women, women with low incomes, and women with less than a high school education were less likely to report having very good or good mouth and teeth condition, having had a dental visit in the previous year, and having had their last dental visit be for preventive care than were non-Hispanic white women or women with higher incomes or more education. In addition, we also found several differences in self-reported oral health conditions and dental visits between pregnant and nonpregnant women.

That a higher percentage of older pregnant women perceived having very good or good oral health than younger pregnant women may be related to a higher proportion of older pregnant women reporting having any dental visit and a preventive dental service in the previous year. This finding suggests that older pregnant women are more conscious of their oral health needs and seek dental care or that they are more likely to have dental insurance than younger pregnant women. Noted differences among younger and older women also could reflect important differences in their socioeconomic status (eg, family income, education). That younger nonpregnant women were more likely to report having very good or good oral health than were older nonpregnant women may be due to the lower cumulative effects of dental disease in younger cohorts (9). Sociodemographic disparities in oral health and dental visits among US women were reported in previous studies (9–13,15). Our results highlight the need to improve use of dental service by US women, especially younger pregnant women, non-Hispanic black and Mexican American women, and women with low family income or low education. In addition, preventive dental care visits are important for improving and maintaining the overall oral health of individuals (16,17). Strategies for reducing oral health disparities and improving access to oral health could include integrating oral health care into overall health care by training nondental health care professionals to screen for oral diseases and reducing financial barriers by raising reimbursement of publicly funded programs so that all women have equal access to oral health services (16–18).

The differences in reported use of dental care between pregnant and nonpregnant women may be related to delaying or postponing dental care until after delivery by both pregnant women and dental professionals to avoid potential adverse pregnancy outcomes (4,13,19,20). However, there is no evidence that dental care, such as dental prophylaxis and tooth scaling, is harmful to a pregnant woman or her developing fetus (3,16,21–23). As discussed in other studies, pregnancy does not preclude dental visits, preventive care, or certain dental treatment (3,16,21,24). On the other hand, postponing dental treatment may complicate existing dental and periodontal conditions. According to a national expert panel convened in 2011,“Preventive, diagnostic, restorative dental treatment is safe throughout pregnancy and is effective in improving and maintaining oral health” (16). In 2013, the American College of Obstetricians and Gynecologists recommended that “. . . an oral health assessment be conducted during the first prenatal visit” and that health professionals “. . . reassure patients that prevention, diagnosis, and treatment of oral conditions are safe during pregnancy” (25). Targeted interventions, including counseling and oral health education during prenatal visits (16,25) to increase knowledge about maintaining good oral health and safety of dental treatments (use of radiographs where clinically indicated, pain medications, and local anesthesia) may improve use of dental services during pregnancy (16). Health care professionals who interact with pregnant women during prenatal visits (eg, obstetricians, nurses, midwives) should be involved in increasing use of dental services during pregnancy (16,18,25). Developing a formal referral process between health care professionals and dental services may help increase awareness of oral health care during prenatal visits (16,25). Nonetheless, socioeconomic barriers such as time constraints and expense may limit access to dental care during pregnancy (4,10,15,19,26–29).

Our estimates of having a dental visit in the previous year among pregnant and nonpregnant women were greater than figures reported by researchers using PRAMS data (10–12) but lower than results reported by researchers using BRFSS data (13). Different survey methods and study designs may account for these differences. In addition, comparisons between surveys should be done with caution, because study design, study period, and methodological approaches to assess dental visits may differ from survey to survey (30). Similarly, our estimates may be greater than those based on PRAMS data because some women classified as pregnant may not have been pregnant when they visited the dentist in the previous year. Our estimates may be lower than those based on BRFSS data because at the time of a study, BRFSS was a home telephone survey, which may not have reached some people with low incomes. People with low incomes in the United States are found to have fewer dental visits in the previous year than people with high incomes (9).

Our study has limitations. Pregnancy status ascertained at the time of interview may not reflect pregnancy status at the time of dental visits in the previous year, leading to potential misclassification of pregnancy status. Self-report bias should also be considered in self-reported data. Despite oversampling, sample sizes were small for some comparisons. Finally, we combined several responses into 1 answer to each of the 3 questions; this may have altered the intention and validity of the original question and may limit the interpretation of the results. Strengths of our study include use of a large, nationally representative sample of the US population and oversampling of pregnant women and population subgroups, which allows for comparisons between pregnant and nonpregnant women and within racial/ethnic groups. We also include more years of data (6 years, 1999–2004) compared with other similar studies (10–13).

Our results show disparities in self-reported oral health conditions and use of dental services among US women overall and between pregnant and nonpregnant women when stratified by sociodemographic characteristics. Results highlight the need to improve use of dental services by US women of childbearing age, especially by young pregnant women, non-Hispanic black and Mexican American women, women with low family incomes, and women with low education levels. Prenatal visits, among other opportunities, could be used to encourage pregnant women to seek preventive dental care during pregnancy.
